# Comparison between *Listeria sensu stricto* and *Listeria sensu lato* strains identifies novel determinants involved in infection

**DOI:** 10.1038/s41598-017-17570-0

**Published:** 2017-12-19

**Authors:** Jakob Schardt, Grant Jones, Stefanie Müller-Herbst, Kristina Schauer, Sarah E. F. D’Orazio, Thilo M. Fuchs

**Affiliations:** 1 ZIEL-Institute for Food & Health, and Lehrstuhl für Mikrobielle Ökologie, Wissenschaftszentrum Weihenstephan, Technische Universität München, Weihenstephaner Berg 3, 85354 Freising, Germany; 2 Department of Microbiology, Immunology, & Molecular Genetics, University of Kentucky, Lexington, Kentucky USA; 3 Lehrstuhl für Hygiene und Technologie der Milch, Tiermedizinische Fakultät, Ludwig-Maximilians-Universität München, Schönleutner Str. 8, 85764 Oberschleißheim, Germany; 4 Friedrich-Loeffler-Institut, Institut für Molekulare Pathogenese, Naumburger Str. 96a, 07743 Jena, Germany

## Abstract

The human pathogen *L*. *monocytogenes* and the animal pathogen *L. ivanovii*, together with four other species isolated from symptom-free animals, form the “*Listeria sensu stricto*” clade. The members of the second clade, “*Listeria sensu lato*”, are believed to be solely environmental bacteria without the ability to colonize mammalian hosts. To identify novel determinants that contribute to infection by *L. monocytogenes*, the causative agent of the foodborne disease listeriosis, we performed a genome comparison of the two clades and found 151 candidate genes that are conserved in the *Listeria sensu stricto* species. Two factors were investigated further *in vitro* and *in vivo*. A mutant lacking an ATP-binding cassette transporter exhibited defective adhesion and invasion of human Caco-2 cells. Using a mouse model of foodborne *L. monocytogenes* infection, a reduced number of the mutant strain compared to the parental strain was observed in the small intestine and the liver. Another mutant with a defective 1,2-propanediol degradation pathway showed reduced persistence in the stool of infected mice, suggesting a role of 1,2-propanediol as a carbon and energy source of listeriae during infection. These findings reveal the relevance of novel factors for the colonization process of *L. monocytogenes*.

## Introduction

*Listeria monocytogenes* is a Gram-positive, facultative anaerobic, non-sporulating, rod-shaped bacterium^1^. The genus *Listeria* belongs to the phylum of Firmicutes, which is composed of Gram-positive bacteria with low GC-content (36–42%) and also includes the genera *Bacillus*, *Clostridium*, *Enterococcus*, *Streptococcus*, and *Staphylococcus* ^2,3^. *L. monocytogenes* is ubiquitous in nature and has been isolated from a variety of ecological niches, such as soil, vegetation, water, and feces^4^. It is a saprophyte that can live on decaying plant material. This prevalence in the environment is promoted by its ability to adapt to salt, acid, and temperature stresses. It can tolerate wide ranges of pH (pH 4.5-9.0) and temperature (0 °C-45 °C) and high concentrations of salt (up to 10% NaCl)^5^. In addition, it can form biofilms^6^. The persistence of certain *L. monocytogenes* strains in processing equipment poses a major challenge for the food industry^7^.

*L. monocytogenes* is the causative agent of the foodborne disease human listeriosis. Consumption of contaminated raw and industrially processed foods such as milk and other dairy products, meat products, vegetables, seafood, and ready-to-eat food is the main cause of infection^1^. Clinical symptoms include gastroenteritis, meningitis, meningoencephalitis, septicemia and prenatal infection^8^. Listeriosis has a high mortality rate of up to 20–30% regardless of early antibiotic treatment, and infants, elderly and immunocompromised individuals are the main risk groups^9^. Upon ingestion, the pathogen encounters a variety of stressful conditions during the gastrointestinal passage, including acidic and osmotic stresses. It is believed that these physiological stresses serve as a signal for priming of the cell for invasion and an intracellular lifestyle^10,11^. For example, acidic and osmotic stresses during passage through the stomach and small intestine trigger the expression of sigma factor B (σ^B^), which induces several stress response- and virulence-associated genes^12,13^. σ^B^ works synergistically with the positive regulatory factor A (PrfA), a thermo-regulated transcription factor active at 37 °C (the body temperature of the host) as well as at ambient temperatures upon induction by low pH^14^; this transcription factor controls expression of the main virulence genes^15^.

The genus *Listeria* currently consists of 17 species. The only other pathogenic member besides *L. monocytogenes* is *L. ivanovii* ^16^, an organism that rarely infects humans^17,18^, but frequently causes listeriosis in ruminants^19,20^. Together with *L. marthii* ^21^, *L. innocua*, *L. welshimeri*, and *L. seeligeri*, these two species form the “*Listeria sensu stricto*” group^22^, one of two distinct clades in the genus *Listeria*. All members of this clade (clade I) have been found in feces or the gastrointestinal tract of symptom-free animals, as well as in food of animal origin^23–27^, suggesting a specific interaction of these species with mammalian hosts. Clade II, the “*Listeria sensu lato*” group, contains the species *L. fleischmanni* 28, *L. weihenstephanensis* ^29^, *L. rocourtiae* ^30^, *L. aquatica*, *L. cornellensis*, *L. riparia, L. floridensis, L. grandensis* ^31^, *L. grayi, L. newyorkensis, and L. booriae* ^32^, which have been isolated from food-associated surfaces or the environment.

While the systemic phase of *L. monocytogenes* infection and the factors involved are well characterized, colonization of the gastrointestinal tract is still underinvestigated. Given that all *sensu stricto* strains have been found in the gut, but only two strains have been associated with human disease, we postulated that there may be bacterial genes encoded within clade I that promote growth or survival in the mammalian gastrointestinal tract. To identify novel listerial genes that could be involved in adhesion to the mucosal epithelium, metabolism, or chemotaxis and motility, we compared the genomes of the *Listeria sensu stricto*-group with the genomes of the environmental strains belonging to the *Listeria sensu lato* group. A total of 151 gene products were identified as being encoded by all *sensu stricto* strains, but absent from the strains of the *sensu lato* group. These factors that possibly contribute to the interaction of listeriae with mammals include the flagellum, the metabolic capabilities to utilize ethanolamine and 1,2-propanediol (1,2-PD), and a set of mainly functionally unknown proteins involved in regulation and transport processes. A putative transporter and the 1,2-PD degradation pathway were tested here for their function and their role during infection by *L. monocytogenes*.

## Results

### *Listeria* phylogenetic groups exhibit differences in colonization ability after oral infection in female BALB/c mice

**Figure 1:**
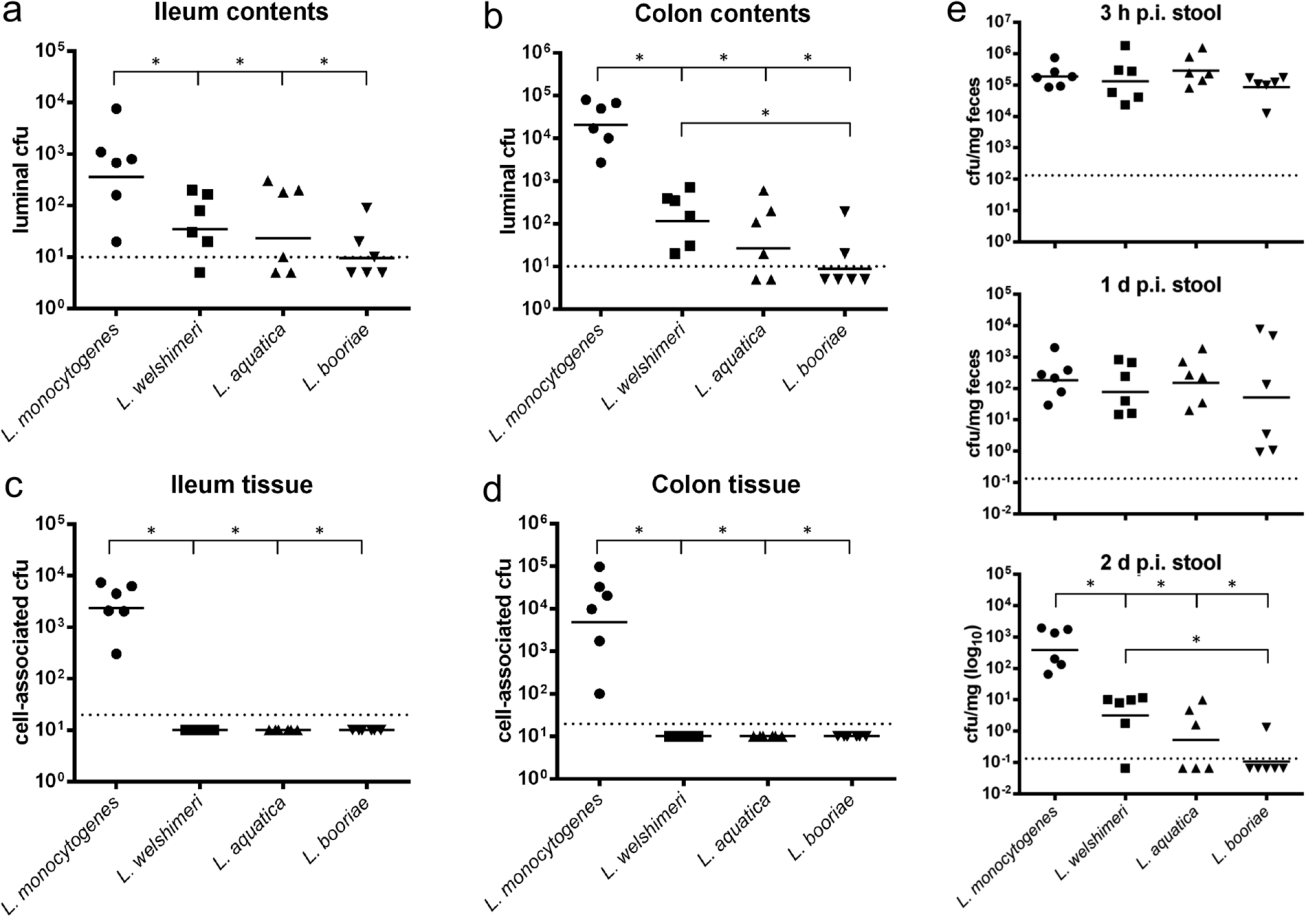
Members of the two listerial phylogenetic groups exhibit different cell numbers in the ileum and colon lumen. Female BALB/c mice were orally infected with 4–9 × 10^8^ cfu of the *Listeria sensu stricto* species *L. monocytogenes* (⚫) and *L. welshimeri* (■), as well as of the *Listeria sensu lato* species *L. aquatica* (▲) and *L. booriae* (▼). After 2 days, cfu numbers for each species were determined in the ileum (**a**) and colon lumen (**b**), as well as in the ileum (**c**) and colon tissue (**d**). Stool samples (**e**) were collected at 3 h p.i., 1 day p.i. and 2 days p.i., and the number of cfu per mg feces was calculated. Symbols represent values for individual mice, while horizontal lines indicate the mean value that was pooled from two separate experiments (n = 3 mice per group). Dashed lines represent the detection limit for each sample. Statistical significance was assessed using two-tailed student’s t-test with Welch’s correction.

To analyze the proliferation of different species from the *Listeria sensu stricto* and *Listeria sensu lato* groups in the gastrointestinal tract and in other organs, female BALB/c mice were orally infected with 4–9 × 10^8^ cell forming units (cfu) of the *Listeria sensu stricto* species *L. monocytogenes* and *L. welshimeri*, as well as the *Listeria sensu lato* species *L. aquatica* and *L. booriae*. The number of luminal *Listeria* in the ileum and colon was determined 2 days post infection (p.i.) (Fig. 1a and b). There were significantly lower cfu numbers of the three non-pathogenic species in both compartments in comparison with the cfu numbers of *L. monocytogenes* strain EGDe. The cfu values of *L. aquatica* and *L. welshimeri* were similar in the ileum. *L. booriae* was barely detectable in both compartments and showed significantly lower mean values in comparison to *L. welshimeri* in the colon, but not in the ileum.

The number of tissue-associated bacteria, that is, in the mucus, the epithelial layer, or the lamina propria (LP) of the gastrointestinal tract, was also analyzed (Fig. 1c and d). While *L. monocytogenes* was found in high numbers of 2.5-6.9 × 10^3^ cfu, the three other species were not detectable in these tissues, confirming their non-pathogenic behavior as reported previously for *L. welshimeri* in mice after intravenous injection^33^. As expected, *L. monocytogenes* could also reach the mesenteric lymph nodes and spread to the liver and spleen of infected mice (Supplementary Fig. 1).

The cfu/mg feces from stool samples taken 3 h p.i., 1 day p.i., and 2 days p.i. of the same mice were additionally analyzed. No significant difference in the cfu/mg feces was observed either immediately after infection or within 24 h of infection. In all cases, the number of cfu decreased 1000-fold, suggesting a species-independent initial clearance of listeriae shed in the feces. After an additional 24 h, *L. monocytogenes* persisted in stool pellets, but the number of cfu decreased significantly for the three non-pathogenic species (Fig. 1e). This effect was more pronounced in the *Listeria sensu lato* species. For example, for half of the mice fed *L. aquatica*, no cfu were detected, and only 1 of 6 mice fed *L. booriae* were still shedding *Listeria*.

### Genome comparison between *Listeria sensu stricto* and *Listeria sensu lato* species reveals candidate genes for infection

The findings described above prompted us to identify as yet unknown genes associated with gut colonization by a genome sequence comparison of 16 out of the 17 known *Listeria* species type strains (all except *L. grayi*). For this purpose, we calculated the percentage of predicted amino acid sequence identity for each *L. monocytogenes* protein against the proteins of the other 15 species within both clades. Gene products with at least 70% sequence homology amongst the *Listeria sensu stricto* species were chosen for further analysis, and filtered against gene products with less than 30% sequence homology amongst the *Listeria sensu lato* species. Although homology of >40% is suggestive of functional homologs^34^, we chose the more stringent threshold to focus on the most robust candidate genes. The resulting Table 1 comprises 151 gene products that are present in all clade I species and absent (or significantly divergent) in clade II species. A majority of the gene products are involved in flagellar biosynthesis and the cobalamin (vitamin B_12_)-dependent utilization of ethanolamine and 1,2-PD. The gain of these clusters has already been described as an evolutionary event that contributed to the taxonomic definition of clade I^35^. Motility is well known to contribute to successful infection by *L. monocytogenes* ^36–40^. Many candidate genes are involved in cell wall and membrane biogenesis, transport processes, transcription, and signaling (Table 1). There is experimental evidence that many of the genes specific for listeriae of clade I contribute to virulence properties, thus validating our approach: Two of the factors involved in peptidoglycan biosynthesis, Lmo0703 and Lmo0717, have been demonstrated to be controlled by the listerial virulence regulators DegU^41^ and MogR^37^. Among the transporters, we listed SvpA (Lmo2185) and Lmo2186 with putative heme transport capacity. SpvA is a listerial virulence factor that promotes escape from phagosomes of macrophages^42^ and/or enables crossing of the digestive barrier^43^. The ATPase synthase encoded by lmo0090-lmo0093 is known to play a role in intracellular survival^44^, and deletion of the gene *pdeD* contributes to a decrease in listerial invasiveness in enterocytes^45^. Because of their predicted, but not experimentally evaluated function *in vitro* or *in vivo*, we chose the putative ATP-binding cassette (ABC) transporter Lmo1131-1132 and the 1,2-PD utilization genes from Table 1 for further functional and infection experiments.

**Table 1:** Genes unique to *Listeria sensu stricto* strains and absent in *Listeria sensu lato* strains.

gene name/lmo number	Function, protein name, or protein homology/similarity to	COG number	Gene name/lmo number	Function, protein name, or protein homology/similarity to	COG number
**Metabolism**			**Miscellaneous**		
lmo0661	Carboxymuconolactone decarboxylase family protein involved in protocatechuate catabolism	COG0599	lmo0090, 0091, 0093	ATP synthase α-/γ-chain, ε-subunit; upregulated in Caco-2 cells and relevant for intracellular replication^44,62^	COG0056, 0224, 0355
*cobU*-*pduO* (lmo1141-1142, 1146-1148, 1169, 1190-1199,1203-1209)	Cobalamin biosynthesis	COG2087	*pdeD* (lmo0111)	c-di-GMP-specific phosphodiesterase, EAL domain	COG2200
*pduS*-*pduQ* (lmo1143-1145, 1151-1171)	1,2-PD degradation pathway	COG4656	lmo0368	Putative Nudix hydrolase YfcD catalyzing the hydrolysis of nucleoside diphosphates	COG1443
*eutA*-*eutQ* (lmo1174-1187)	Ethanolamine utilization pathway	COG4819	lmo0481	Myosin-Cross-Reactive Antigen, oleate hydratase; upregulated *in vivo* ^87^	COG4716
**Cell motility and chemotaxis**			lmo0511	Glutamine amidotransferase; DegU regulated	COG0518
*fliN*–*fliS* (lmo0675-lmo0718)	Flagellar biosynthesis	—	lmo0617	Putative lipoprotein with DUF4352 domain, immunoprotective extracellular protein0212	—
lmo0723, lmo1699	Methyl-accepting chemotaxis proteins	COG0840	lmo0625	Lipolytic protein GDSL family	COG2755
**Cell wall/membrane/envelope biogenesis**			lmo0635	2-Haloalkanoic acid dehalogenase with phosphatase activity	COG1011
lmo0703	UDP-N-acetylenolpyruvoylglucosamine reductase involved in peptidoglycan biosynthesis; regulated by DegU and MogR^88,89^	COG1728	lmo1415	Hydroxymethylglutaryl-CoA synthase involved in isopentenyl pyrophosphate synthesis via the mevalonate pathway; possibly influences Vγ9/Vδ2 T cells^90^	COG3425
lmo0717	Murein transglycosylase with N-acetyl-D-glusoamine binding site, SLT family; DegU and MogR regulated^88,89^	COG0741	lmo1638	Microcin C7 self-immunity protein MccF; its absence results in listerial accumulation in the liver^91^	COG1619
lmo0724	Peptidase	COG4990	lmo2424	Thioredoxin	COG3118
**Transporter**			**No orthologues**		
lmo0269	Oligopeptide transport system permease protein	COG1173	lmo0615	Hypothetical protein	—
lmo0987	ABC transporter, permease protein, induced in CodY mutant^92^; related to CylB of *Streptococcus agalactiae* involved in hemolysin production	COG1511	lmo0622	Hypothetical protein	—
lmo1131, 1132	ABC transporter, ATP-binding/permease protein	COG4988	lmo0657	Hypothetical protein	—
lmo2181	NPQTN specific sortase B; surface protein transpeptidase involved in anchoring of SvpA and Hbp1	COG4509	lmo0793	Putative transport protein	COG1811
*svpA*/*hbp2*(lmo2185), *hbp1* (lmo2186)	NPQTN cell wall anchored proteins with similarity to IsdA, IsdC of *Staphylococcus aureus* ^93^; heme-binding NEAT domain associated with iron/heme transport^94^; Fur and MecA regulated virulence factor^42,43^	COG5386	lmo0819	Hypothetical protein	—
lmo2669	Type IV ABC-transporter, upregulated under anaerobic conditions^80^	COG4905	lmo1626	Hypothetical protein	—
**Transcription**			lmo1779	Hypothetical protein	—
lmo0212	Acetyltransferase, GNAT family	COG0456	lmo2063	Hypothetical protein	—
lmo1309, 1310	Co-activators of prophage gene expression IbrB, IbrA	COG1475, COG3969	lmo2065	Hypothetical protein	—
lmo1311	SNF2 family domain protein	COG0553	lmo2066	Hypothetical protein	—
lmo2234	Sugar phosphate isomerase	COG1082	lmo2169	Hypothetical protein	—
			lmo2432	Hypothetical protein; Fur-box^43^	—
			lmo2803	Hypothetical protein	—
			lmo2843	Hypothetical protein	COG5279

### The operon lmo1131-1132 encoding a putative ABC transporter facilitates adhesion and invasion of *L. monocytogenes* in Caco-2 cells

**Figure 2:**
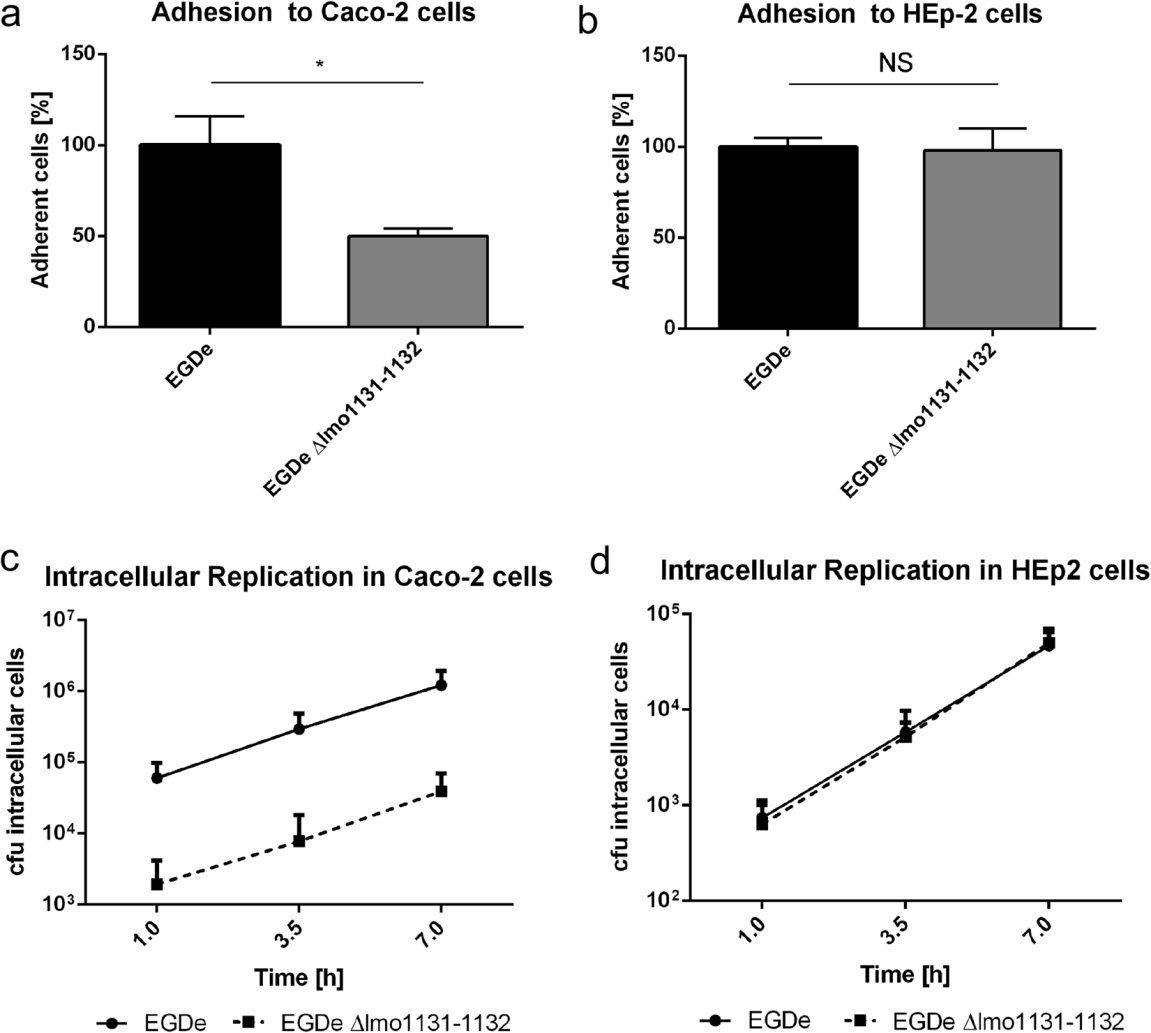
Adhesion to and invasion of Caco-2 and HEp-2 cells by a transporter mutant. The percentage of adherent cells of *L. monocytogenes* EGDe and EGDe Δlmo1131-1132 to approximately 2.5 × 10^5^ eukaryotic Caco-2 cells (**a**) or HEp-2 cells (**b**) was analyzed for a MOI of 10. The number of adherent EGDe cells was set to 100%. (**c**,**d**) The percentage of invasive and replicating cells of *L. monocytogenes* EGDe and EGDe Δlmo1113-1132 was determined. Approximately 2.5 × 10^5^ eukaryotic Caco-2 or HEp-2 cells were infected with a MOI of 10, and the numbers of intracellular bacterial cells were determined 1, 3.5 and 7 h p.i. Error bars indicate the standard deviation of three biologically independent experiments including technical replicates. Statistical significance was assessed using two-tailed student’s t-test with Welch’s correction; NS, not significant.

According to the Basic local alignment search tool on the NCBI homepage, the operon lmo1131-1132 encodes a putative ABC transporter. Both proteins Lmo1131 and Lmo1132 contain an ABC transporter-like transmembrane domain as well as a nucleotide binding domain. For further analysis of the functional role of the operon lmo1131-1132, we constructed the in-frame deletion mutant *L. monocytogenes* EGDe Δlmo1131-1132 and tested adhesive and invasive capabilities in cell culture assays using Caco-2 (human colon carcinoma) cells and HEp-2 (human larynx squamous cell carcinoma) cells. Adhesion to Caco-2 cells was reduced in the deletion mutant compared with that of the parental strain EGDe by a factor of 2 (Fig. 2a). In contrast, cell culture assays with HEp-2 cells revealed no significant adhesion differences between EGDe and EGDe Δlmo1131-1132 (Fig. 2b), suggesting a specific role of the ABC transporter in the interaction with Caco-2 cells. We also performed a gentamycin protection assay to test the invasion and the intracellular replication properties of the deletion mutant in Caco-2 and HEp-2 cells. Given that the number of intracellular cells detected 1 h after cell culture infection is a measure of the capacity of a strain to enter cells, we observed a more than 20-fold reduction of invasion of Caco-2 cells, but not of HEp-2 cells, by EGDe Δlmo1131-1132 compared with that of the parental strain (Fig. 2c and d). The intracellular replication rates of EGDe [doubling time T_d_ (EGDe) = 83 min] and EGDe Δlmo1131-1132 [T_d_ (EGDe Δlmo1131-1132) = 83 min] however, were identical in the Caco-2 cells, indicating a specific role of the putative transporter in adhesion and/or invasion of this cell type.

### Transcription of lmo1131-1132 shows oxygen- and temperature-dependent regulation

**Figure 3:**
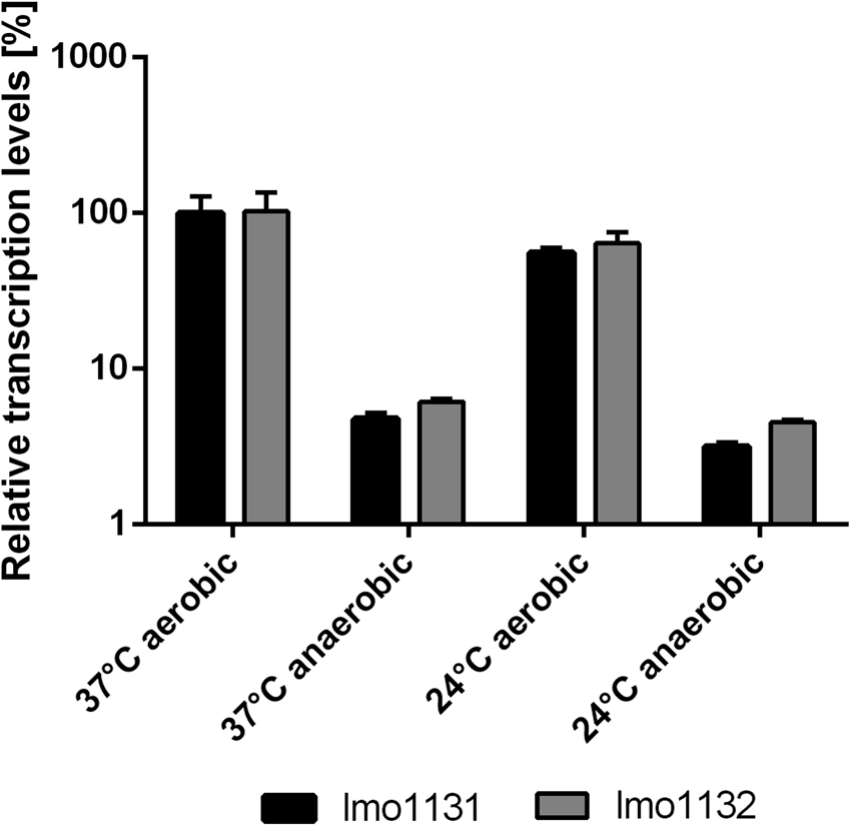
Temperature- and oxygen-dependent transcription of lmo1131 and lmo1132. Relative transcription of genes lmo1131 (black bars) and lmo1132 (gray bars) for the conditions 37 °C anaerobic, 24 °C aerobic, and 24 °C anaerobic was compared to that at 37 °C aerobic (preassigned as 100% gene expression for lmo1131). The results were calculated using the 2^−ΔΔCT^ method^95^ and lmo1759 (*pcrA*) was used as a reference gene for normalization. Error bars indicate the standard error of three biologically independent experiments including technical duplicates for each condition.

We then examined the transcription of lmo1131 and lmo1132 in *L. monocytogenes* EGDe at 24 °C and 37 °C under both aerobic and anaerobic growth conditions via quantitative real time (qRT)-polymerase chain reaction (PCR) (Fig. 3). The two transporter genes exhibited similar transcription levels under all four conditions, suggesting transcription of the operon from a common promoter located upstream of lmo1131. Setting the transcriptional level of both genes at 37 °C with oxygen as 100%, we observed a decrease of the lmo1131 and lmo1132 mRNA levels at 37 °C to 4.77% and 6.15%, respectively, upon oxygen depletion. At 24 °C, the transcription of the transporter genes was reduced to 55.80% and 64.02%, respectively, under aerobic conditions and to 3.18% and 4.53% in the absence of oxygen.

### Lack of lmo1131-1132 leads to attenuated colonization of BALB/c mice

**Figure 4:**
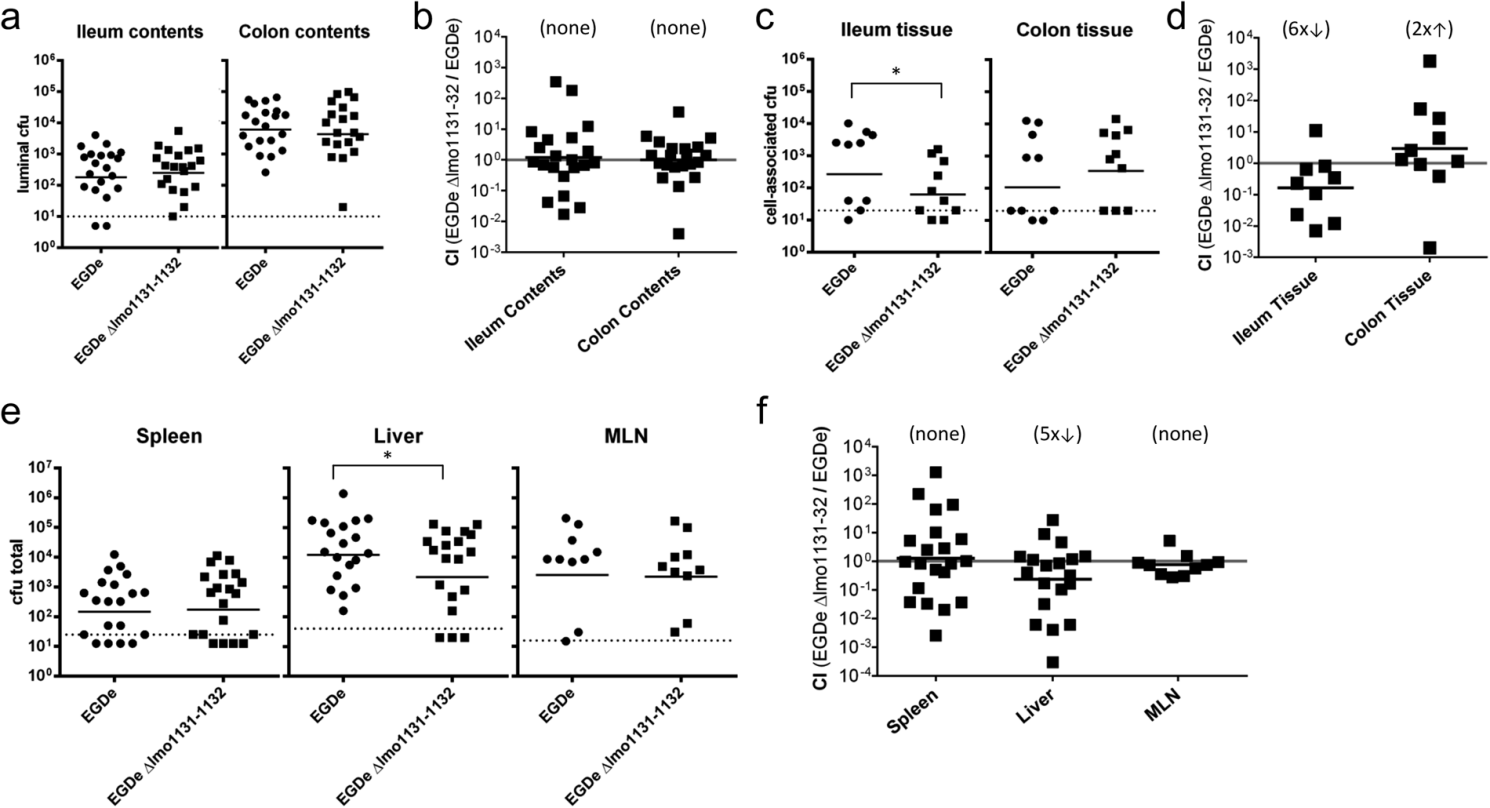
Deletion of lmo1131-1132 leads to attenuated colonization of BALB/c mice. Female BALB/c mice were orally infected with a 1:1 ratio of *L. monocytogenes* EGDe and EGDe Δlmo1131-1132 for a total inoculum of 1 × 10^9^ cfu. After 2 days, the cfu of luminal (**a**) or cell-associated (**c**) strains in the ileum and colon were determined using kanamycin- (EGDe) or erythromycin (mutant)-containing plates, and the CI was calculated (**b,d**). (**e**) The cfu numbers of both strains in the spleen, liver and MLNs are shown. The numbers of EGDe (circles) or EGDe Δlmo1131-1132 (squares) recovered in each mouse after co-infection are depicted. Solid horizontal lines indicate mean values, which were pooled from at least two separate experiments, while dashed lines represent the detection limit for each sample. (**f**) The CIs of the strains recovered from the organs are depicted. The geometric mean for each group was compared to the theoretical value of 1.0, and the fold change difference is indicated in parentheses. Statistical significance was assessed using two-tailed student’s t-test with Welch’s correction.

To verify the relevance of the candidate genes for the *in vivo* infection process, a food-borne transmission model in mice was used to enable study of the gastrointestinal phase of listeriosis^46^. We conducted co-infection experiments with female BALB/c mice aged 6-8 weeks using a 1:1 ratio of EGDe and its mutant EGDe Δlmo1131-1132 lacking the putative transporter genes. The strains were chromosomally tagged with pIMC3kan (EGDe) and pIMC3ery (mutant). Mice were fed a total of 1 × 10^9^ cfu/mouse and the number of *Listeria* in the lumen of either the ileum or the colon was analyzed 2 days p.i. (Fig. 4a and b). The colon contained 25-fold more *L. monocytogenes* than the ileum, as was previously reported by others^46,47^. However, there was no significant difference in the ratio of the two strains recovered from the intestinal contents of either tissue.

We then assessed the numbers of cfu associated with the flushed tissue, which includes *L. monocytogenes* trapped in the mucus layer, bacteria adhered to or inside intestinal epithelial cells (ECs) and bacteria that have reached the underlying LP. In both the ileum and the colon, a bimodal distribution was observed, with little or no *L. monocytogenes* recovered in approximately half of the mice, and up to 10^4^ cfu in the other half (Fig. 4c). In the ileum, the average number of cfu for the latter group was six-fold higher for wildtype EGDe compared with the mutant. Overall, the competitive index (CI) revealed that the wild type strain outcompeted the mutant strain six-fold in the ileum (Fig. 4d). Interestingly, there was no significant difference between EGDe and EGDe Δlmo1131-1132 in the colon.

To further investigate this difference in intestinal colonization, the gut tissues of infected mice were fractionated, and the number of cfu in either the mucus layer, ECs or the LP were determined (Supplementary Fig. 2a). The number of wild type and EGDe Δlmo1131-1132 in the mucus and the epithelial layer of the ileum did not differ significantly. However, wild type *L. monocytogenes* outnumbered the deletion mutant by five-fold in underlying LP of the ileum, similar to what was observed for whole tissue. As expected, there were no differences in the competitive indexes for any of the colon fractions. To assess the systemic spread of both strains, we also determined the bacterial load in the mesenteric lymph nodes (MLN), spleen and liver 2 days p.i. As shown in Fig. 4e and f, five-fold fewer EGDe Δlmo1131-1132 were recovered from the liver compared to the wild type strain. There was no significant difference between the two strains in the MLN or the spleen. To test whether dissemination of the transporter mutant to peripheral organs was impaired or if the mutant strain had a specific growth defect in the liver, mice were intravenously injected with a 1:1 mixture of EGDe and EGDe Δlmo1131-1132, and the total cfu in liver and spleen were determined 2 days p.i. Approximately equal numbers of both strains were recovered from the liver when the transmission route bypassed the gut (Supplementary Fig. 2b). Together, these data suggest that the transporter Lmo1131-1132 is involved in efficient translocation across the intestinal mucosa in the small intestine, but not the large intestine.

### The *pduD* gene of *L. monocytogenes* is required for growth with 1,2-PD

**Figure 5:**
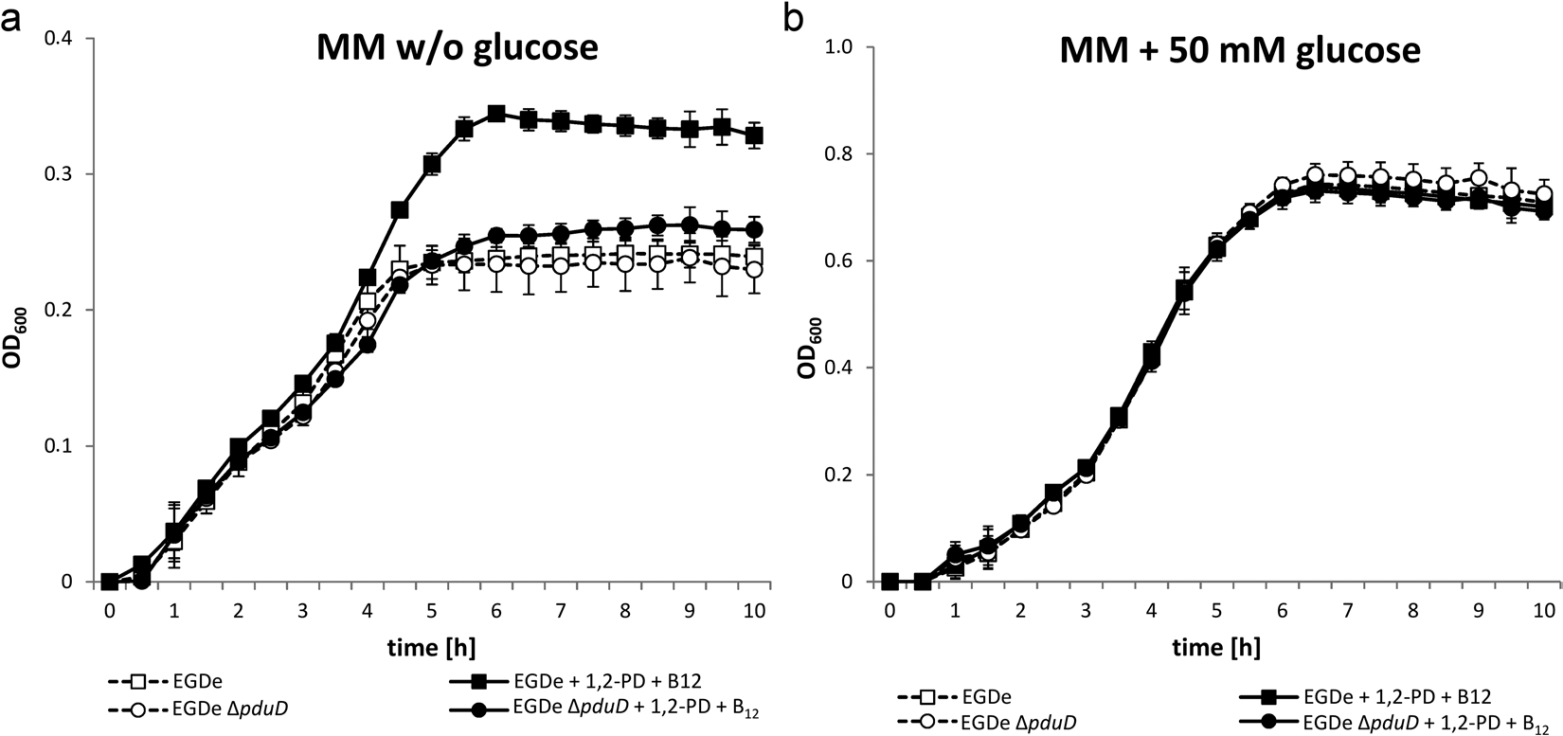
Improved growth of *L. monocytogenes* by addition of 1,2-PD. Growth curves of *L. monocytogenes* EGDe (□) and *L. monocytogenes* EGDe Δ*pduD* (○) in MM and 0.5% (w/v) yeast extract without glucose (**a**) or with 50 mM glucose (**b**) cultivated at 37 °C under anaerobic conditions. MM was supplemented with 10 mM 1,2-PD and 25 nM cobalamin (filled symbols) or not supplemented (open symbols). OD_600_ was measured at the indicated intervals using Bioscreen C. Growth curves depict the calculated mean value of three independent biological experiments with technical duplicates, while error bars indicate the standard deviation.

Three large gene clusters responsible for the cobalamin-dependent utilization of ethanolamine and 1,2-PD were identified to be specific to the clade *sensu stricto* species of the genus *Listeria* ^22^ (Table 1). These pathways have been demonstrated to play a role in the proliferation of *S*. Typhimurium *in vitro* and *in vivo* ^48–50^, whereas no such experimental data have been available for *L. monocytogenes*. To investigate the role of 1,2-PD as a potential carbon and energy source for *L. monocytogenes*, strain EGDe and its mutant EGDe Δ*pduD*, which lacks a gene essential for 1,2-PD utilization, were grown in minimal medium^51^ (MM) without glucose, but with 0.5% (w/v) yeast extract under anaerobic conditions at 37 °C. No significant differences in the growth behavior of the two strains were observed. However, when we added 1,2-PD and cobalamin, which is an essential cofactor of PduCDE and whose biosynthesis is encoded by the *cob*/*cbi* gene cluster, EGDe grew to an optical density (OD)_600_^max^ = 0.35, whereas the cell density of EGDe Δ*pduD* did not exceed that in the medium without 1,2-PD (Fig. 5a). Therefore, it might be speculated that all nutrients preferred over 1,2-PD are used up during the first 4 h, and that *L. monocytogenes* only then degrades 1,2-PD to reach a higher cell density.

As a control, we compared the growth of both strains in MM containing 50 mM glucose in the absence and presence of 1,2-PD and cobalamin. Under these growth conditions with glucose as an energy source, the addition of 1,2-PD had no significant effect on the cell density of either EGDe or EGDe Δ*pduD* (Fig. 5b), suggesting that 1,2-PD is utilized by *L. monocytogenes* only in the absence of glucose.

### Supplementation with 1,2-PD and/or cobalamin leads to induction of *pdu* and *cob*/*cbi* genes during the stationary phase

**Figure 6:**
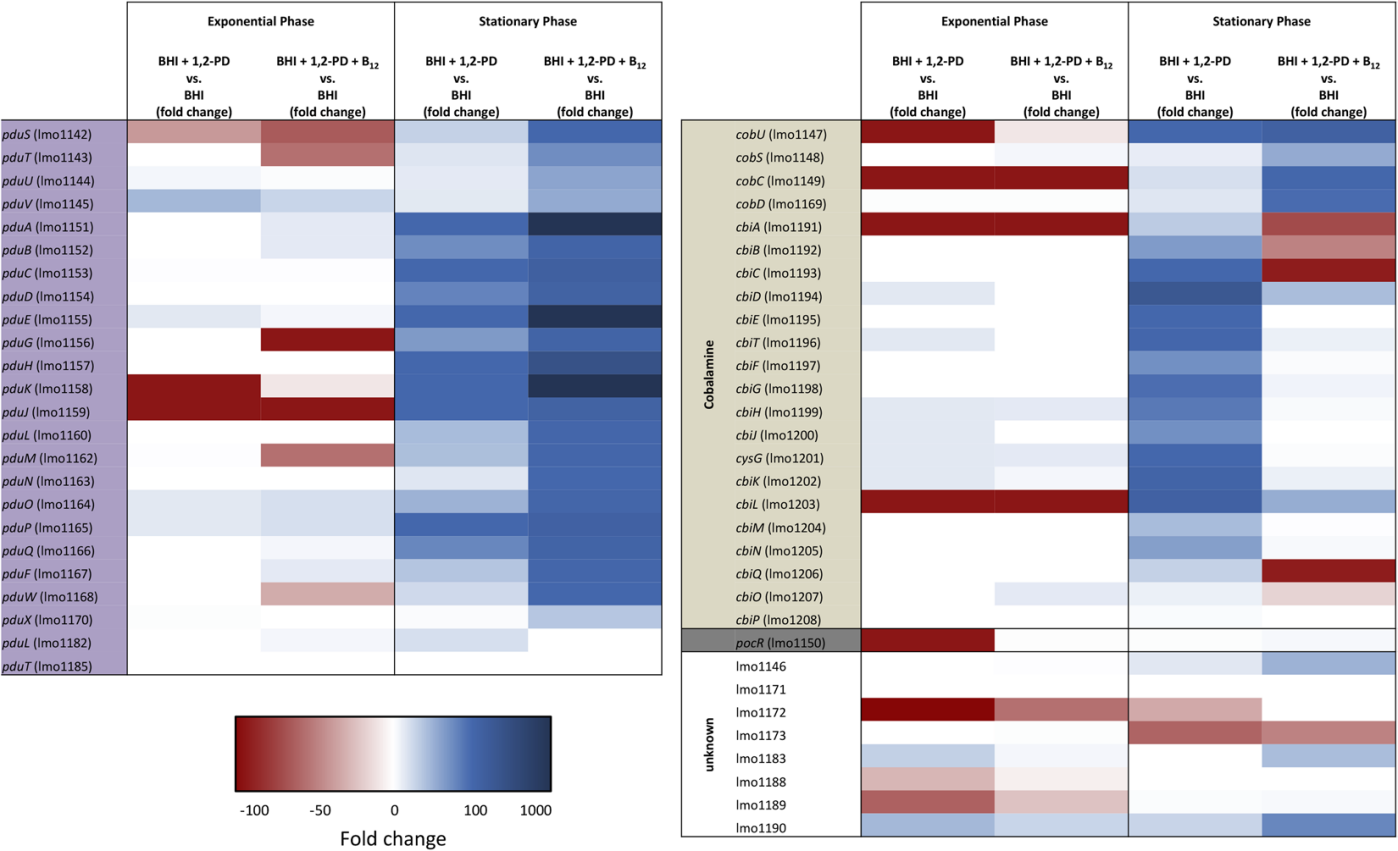
Transcriptome in response to growth with 1,2-PD. *L. monocytogenes* EGDe grown under different conditions was harvested during the exponential and stationary phases and the mRNA expression profile was analyzed via NGS using the Illumina MiSeq seqencing platform. Fold changes of normalized RPKM (reads per kilobase per million mapped reads) values under the conditions BHI with 10 mM 1,2-PD (BHI + 1,2-PD) and BHI with 10 mM 1,2-PD and 25 nM cobalamin (BHI + 1,2-PD + B_12_) in comparison to BHI were calculated for both growth phases. The results were visualized using a three-color scheme with red colors indicating negative and blue colors indicating positive fold changes; white means no change. The color intensity corresponds to the magnitude of fold change. Unknown genes refer to genes located in the region of propanediol, ethanolamine, and cobalamin clusters without any known function.

To investigate the transcriptional response of the *pdu* and the *cob*/*cbi* gene clusters to the presence of 1,2-PD and/or cobalamin, a global transcriptome analysis of *L. monocytogenes* EGDe was conducted via next-generation sequencing (NGS). During growth in brain heart infusion (BHI) medium, in BHI medium with 10 mM 1,2-PD, or in BHI medium with 10 mM 1,2-PD and 25 nM cobalamin, cells were harvested in the mid-logarithmic (exponential) and stationary phases. During the exponential phase, the *pdu* and the *cob*/*cbi* gene clusters showed only weak or no transcription under all three growth conditions. During the stationary phase, transcription of these genes only slightly increased in BHI medium, although levels were still very low (Supplementary Table S1). However, when 1,2-PD was added to the cultures, a strong increase of expression of most *pdu* and *cob*/*cbi* genes in the range from two-fold to 1,605-fold induction was observed (Fig. 6). The transcription of *pdu* genes was even more elevated (up to more than 10,942-fold) when cobalamin was added, and the presence of this cofactor in the medium resulted in repression of the genes responsible for its biosynthesis (Supplementary Table S1), a finding that confirms a regulatory model in which the presence of cobalamin represses its own synthesis post-transcriptionally via stabilization of an mRNA hairpin^52^. The transcription levels of *pocR*, the regulator of both clusters, were only slightly elevated under medium supplemented with 1,2-PD and/or cobalamin. The *eut* genes of the ethanolamine degradation cluster, which are positioned between the *pdu* and *cbi*/*cob* clusters, were not significantly induced under the growth conditions applied (Supplementary Table S1).

The transcriptome data were succesfully validated via qRT-PCR for the selected genes lmo1146, *pocR* (lmo1150), *pduC* (lmo1153), lmo1190 and *cbiA* (lmo1199) in stationary phase (Table 2). In exponential phase, there is a discrepancy between qRT-PCR and NGS results due to the higher sensitivity of qRT-PCR and the overall low number of RPKM values from the NGS (Suppl. Table S1). The results of both the transcriptome analysis and the qRT-PCR are compatible with the observation that the addition of 1,2-PD and cobalamin to *L. monocytogenes* EGDe in MM led to a growth advantage after exponential phase when other nutrients had been used up (Fig. 5a).

**Table 2:** Relative transcription in percent of the genes lmo1146, *pocR* (lmo1150), *pduC* (lmo1153), lmo1190 and *cbiH* (lmo1199) during growth in BHI with 10 mM 1,2-PD (BHI + 1,2-PD), in BHI with 10 mM 1,2-PD and 25 nM cobalamin (BHI + 1,2-PD + B_12_), or in BHI.

Gene		Stationary Phase			Exponential Phase		
		BHI*	BHI + 1,2-PD	BHI + 1,2-PD + B_12_	BHI	BHI + 1,2-PD	BHI + 1,2-PD + B_12_
lmo1146	qRT-PCR	100	1,845	6,664	250	454	578
	NGS	100	1,623	5,038	49	50	90
lmo1150	qRT-PCR	100	421	1,137	84	111	108
	NGS	100	261	566	21	1	48
lmo1153	qRT-PCR	100	9,078	148,856	51	101	205
	NGS	100	12,377	77,536	25	50	45
lmo1190	qRT-PCR	100	20,583	52,177	70	216	288
	NGS	100	2,874	7,905	2	75	45
lmo1199	qRT-PCR	100	7,291	697	536	915	184
	NGS	100	8,724	486	3	50	45

*The transcriptional activity obtained in the stationary phase of growth with BHI was set as 100% for each gene.

### Deletion of *pduD* leads to faster clearance in BALB/c mice

**Figure 7:**
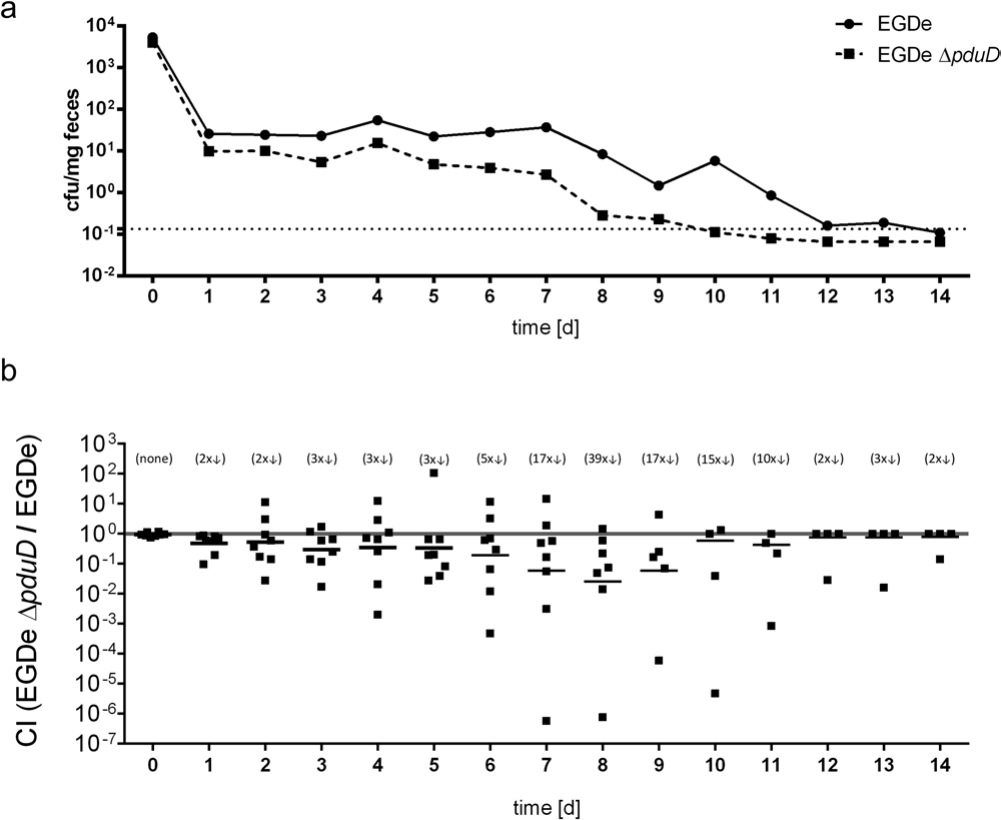
Deletion of *pduD* leads to faster clearance in BALB/c mice. Female BALB/c mice were orally infected with a 1:1 ratio of *L. monocytogenes* EGDe and EGDe Δ*pduD* for a total inoculum of 1 × 10^9^ cfu. Stool samples were collected 3 h p.i. as well as every 24 h up to 16 days and the mean value of cfu per mg feces was calculated (**a**). Symbols represent mean values from two separate experiments (n = 4 mice per group) for *L. monocytogenes* EGDe (circles) and EGDe Δ*pduD* (squares) and dashed line represents the limit of detection. (**b**) CIs depict the ratio of EGDe Δ*pduD*/EGDe. The geometric mean for each group was compared to the theoretical value of 1.0 and the fold change difference is indicated in parentheses.

We hypothesized that the ability to metabolize 1,2-PD would enhance the ability of *L. monocytogenes* to compete with gut microbiota and to persist in the intestinal lumen following oral transmission. To test this, female BALB/c mice were co-infected with a 1:1 ratio of wild type EGDe and an isogenic Δ*pduD* mutant (total of 1 × 10^9^ cfu/mouse) that were differentially tagged with antibiotic resistances. Stools were collected daily for up to 14 days p.i., and the total number of strain per mg of feces was determined. As expected, a significant portion of the inoculum was shed in feces within 3 hours after feeding the mice, and the number of cfu detected 21 hours later had decreased considerably (Fig. 7a). At each time point after that, the average number of Δ*pduD* mutant bacteria recovered was less than the wildtype strain. By 10 days post-infection, the Δ*pduD* mutant had been cleared, but wildtype bacteria continued to be shed in the feces in at least some of the mice for up to 4 more days. Accordingly, the competitive index decreased steadily over the course of the experiment with a maximum difference on day 8 when the wild type bacteria outcompeted the mutant bacteria by nearly 40-fold (Fig. 7b). Thus, the presence of the genes encoding the 1,2-PD utilization pathway prolonged the survival of *L. monocytogenes* EGDe in the mouse gastrointestinal tract.

## Discussion

It is believed that a common ancestor of the *Listeria sensu stricto* group took up a set of virulence genes through horizontal gene transfer, and that these determinants were subsequently lost in the non-pathogenic species of this listerial clade I^53,54^. Other putative evolutionary processes differentiating *Listeria sensu stricto* from *Listeria sensu lato* are the expansion of internalin genes and the acquisition of the metabolically relevant *eut*/*pdu*/*cbi*/*cob* gene cluster as well as of flagellar genes, which were probably taken up via horizontal gene transfer from an ancestor of the *Bacillus cereus* complex^22^. The association of *Listeria sensu stricto* with fecal samples or the gastrointestinal tract of mostly symptom-free animals or food from animal origins^23–27^, a reduced colonization rate of the environmental species *L. fleischmannii* and *L. floridensis* compared to *Listeria sensu stricto* species in fecal samples from wild rodents^55^, as well as the alleviated colonization abilities of the *Listeria sensu lato* species in our BALB/c infection experiments, prompted us to speculate that a genome comparison of clade I versus clade II strains might identify as yet unknown listerial factors that play a role particularly during the gastrointestinal phase of infection.

Mice and other animal model organisms are considerably more resistant to oral infections than humans, and larger inocula need to be administered (10^9^–10^11^ cfu/animal) to cause an intestinal infection^56^. A high species specificity of the surface proteins InlA, which interacts well with ECs of humans or guinea pigs, but not with ECs of mice^57,58^, contributes to inefficient oral transmission. Recent studies also show that competition with gut microbiota may limit opportunities for *L. monocytogenes* to invade the gut mucosa^47,59^. To investigate the relevance of as yet unknown listerial determinants for the gastrointestinal phase of listeriosis in more detail, we used a recently established and highly reproducible feeding model to mimic the natural course of infection^46^ that overcomes the lack of reproducibility encountered by the conventional administration of listeriae by oral gavage.

Gastrointestinal infection is a multifactorial process and, besides the already mentioned stress conditions, metabolism is one of the key factors of pathogenic bacteria that can only survive in the intestine by searching for a specific niche providing sufficient amounts of nutrients^60^. A hallmark of pathogens is therefore their metabolic flexibility during infection. Examples of this for *L. monocytogenes* include the utilization of sugar phosphates and glycerol as alternative carbon sources during intracellular replication^61–63^, the requirement of both thiamine uptake and biosynthesis of thiamine precursors^64^, or the possible exploitation of uncommon nitrogen sources such as ethanolamine or glucosamine^65^. *L. monocytogenes* possesses a 53-kb island harboring genes for the metabolism of 1,2-PD, ethanolamine, and cobalamin, which can be found in all *Listeria sensu stricto* species^22^. These compounds can be found in the gastrointestinal tracts of animals and have been shown to play an important role in *Salmonella* pathogenesis^49,66,67^. It has been discussed whether these gene clusters are also relevant for the virulence of listeriae, particularly for colonization^54^. So far, it has been shown that ethanolamine utilization contributes to intracellular replication in ECs^62^, and that 1,2-PD is metabolized by *L. innocua* ^68^. Transcriptional studies in gnotobiotic mice infected with *L. monocytogenes* showed upregulation of most *pdu* genes during infection compared to growth in BHI^69^. Our study not only demonstrates that *L. monocytogenes* can use 1,2-PD as a nutrient but also provides strong experimental evidence that the ability of *L. monocytogenes* to catabolize 1,2-PD contributes to its persistence and proliferation during gastrointestinal infection. This is in line with the finding that *S*. Typhimurium expansion is driven by 1,2-PD and that its utilization requires not only intestinal inflammation, but also the presence of commensal bacteria^50^. These are assumed to provide 1,2-PD, for example, by the fermentation of fucose that has been cleaved from the mucosal glycans by commensal bacteria^70,71^.

Toledo-Arana and colleagues showed that lmo1131 but not lmo1132 expression is decreased in intestinal *L. monocytogenes* of gnotobiotic mice compared with that under BHI growth conditions^69^. Their reduced transcription at 24 °C and under anaerobic conditions suggests a role in an early stage of infection. A typical prokaryotic ABC transporter is composed of two hydrophobic transmembrane domains (TMDs) and two water soluble nucleotide binding domains (NBDs) at the cytosolic side of the cell membrane. An ABC transporter can be composed of four separate polypeptides, or two identical NBDs and/or TMDs can be present. NBDs and TMDs can also be fused together thus making up either the complete transporter from a single polypeptide or from two homo- or heterodimeric polypeptides^72^. Lmo1131 and Lmo1132 seem to belong to the group of “half transporters”, with two heterodimeric halves, each containing one NBD and one TMD. In addition to import functions, ABC transporters are involved in sorting molecules to the outer membrane. These molecules include lipoproteins, polysaccharides or fimbriae that might contribute to the interaction with mammalian cells^73,74^. Examples from human pathogens are the export of a coat protein from EAEC contributing to bacterial dispersion, the polysaccharide-dependent adhesion of *Kingella kingae* to human ECs, and the fimbriae-mediated adhesion of *Streptococcus parasanguis* ^75–77^. Another example is protein F as part of an ABC transporter from *Haemophilus influenzae* whose N-terminus promotes binding to ECs^78^.

To conclude, non-pathogenic *Listeria* species have been investigated here for the first time in a mouse model for oral infection, and the results suggest differences in the colonization ability of *Listeria sensu stricto* and *Listeria sensu lato* members. Novel *Listeria sensu stricto*-specific factors involved in the colonization of the gastrointestinal tract were identified, whereas as the *Listeria sensu lato* species seem to be less adapted to the conditions of the gastrointestinal tract. Because the non-pathogenic *Listeria* species of the *sensu stricto* group including *L. welshimeri* are equipped with these determinants, their potential to occupy niches of the pathogenic species has been underestimated.

## Methods

### Bacterial strains, plasmids, cell lines and growth conditions

The bacterial strains, cell lines, and plasmids used in this study are listed in Supplementary Table S2. *E. coli* was grown in Luria-Bertani broth, while *L. monocytogenes* EGDe was cultivated in BHI or MM^51^ at 37 °C or 24 °C. If appropriate, the media were supplemented with the following antibiotics: erythromycin (300 μg/ml for *E. coli* or 10 μg/ml for *L. monocytogenes*), kanamycin (50 µg/ml), and chloramphenicol (10 µg/ml). For solid media, 1.5% agar (w/v) was added. Human colon ECs (Caco-2 cells, ATCC HTB-37) and human larynx squamous cell carcinoma cells (HEp-2 cells, ATCC CCL-23) were received from the American Type Culture Collection and were cultured at 37 °C and 5% CO_2_ in Dulbecco’s Modified Eagle Medium (Biochrom KG, Berlin, Germany) supplemented with 10% fetal calf serum (Pan Biotech, Aidenbach, Germany). For RNA isolation, 50.5 ml of BHI was inoculated with 0.5 ml of a *L. monocytogenes* overnight culture and incubated at 37 °C or 24 °C with shaking (150 rpm). Aerobic growth in broth was conducted in Erlenmeyer flasks with constant shaking; anaerobic growth was performed in sealed Falcon tubes. Growth was monitored by measuring OD_600_ with a Lambda Bio + spectrophotometer (Perkin Elmer, Waltham, MA, USA). If appropriate, 3.5 ml of BHI supplemented with 10 mM 1,2-PD (Sigma-Aldrich, Taufkirchen, Germany) and/or 25 nm cobalamin (Applichem, Darmstadt, Germany) in a 15-ml glass tube was inoculated with 0.25 ml of an *L. monocytogenes* overnight culture and incubated at 37 °C with shaking.

### Standard procedures

DNA manipulations and isolation of chromosomal DNA were performed in accordance with standard protocols^79^ and following the manufacturer’s instructions. GeneRuler^TM^ 1 kb DNA Ladder from Thermo Scientific (Waltham, MA, USA) was used as a marker for DNA analysis. A Bio-Rad Gene pulser II was used for electroporation. PCR was carried out with Taq polymerase and with 100-400 ng of chromosomal DNA or an aliquot of a single colony resuspended in 50 µl of H_2_O as template. Quantitative real-time (qRT)-PCR and whole bacterial RNA isolation were perfomed as described recently^80^. Transcription of the housekeeping gene 16 S rRNA and lmo1759 (*pcrA*) was used for normalization. The oligonucleotides used in this study are listed in Supplementary Table S3. For listerial gene annotation, the *Listeria* homepage of the Institut Pasteur (http://genolist.pasteur.fr/ListiList/) was used.

For genome comparison, type strain genomic sequences of the 16 *Listeria* species were downloaded from NCBI GenBank. Sequences were uploaded to the RAST server^81^ and functionally annotated. *L. grayi* was excluded from this comparison since it is most closely related to *Listeria sensu stricto*, although it belongs to the *Listeria sensu lato group*.

In-frame gene deletions were performed as described recently^62^. Briefly, two flanking fragments of ~1000 bp were amplified from chromosomal DNA derived from the strain EGDe using the oligonucleotides indicated in Supplementary Table S3 and ligated *via* the introduced *Bgl*II sites. Following nested PCR and using the ligation mixture as a template, the resulting fragment was cloned into pLSV101 *via Sal*I and *Xma*I. Following transformation of the resulting plasmids, erythromycin-resistant EGDe harboring the chromosomally integrated plasmid were selected upon incubation at 42 °C. Cointegrates were resolved, erythromycin-sensitive clones were screened by PCR, and the deletion sites were sequenced to identify the respective mutant strains.

### Transcriptome analysis

Whole-transcriptome RNA library preparation was performed as described recently^82^. Briefly, RNA was extracted, ribosomal RNAs were depleted, and RNA was fragmented via sonication. After dephosphorylation and rephosphorylation, TruSeq Small RNA Sample Kit (Illumina, Munich, Germany) was used, and the resulting cDNAs were size-selected. Libraries were then diluted and sequenced on a MiSeq sequencer (Illumina, Munich, Germany) using a MiSeq Reagent Kit v2 (50 cycles), resulting in 50 bp single-end reads. Illumina FASTQ files were mapped to the reference genome of *L. monocytogenes* EGDe (GenBank: NC_003210) using Bowtie for Illumina implemented in Galaxy^83,84^. SAM files were converted to BAM files and indexed. Artemis^85,86^ was used to visualize and calculate the number of reads mapping on each gene. Gene counts of each library were normalized to the smallest library in the comparison and RPKM (reads per kilobase per million mapped reads) values were calculated. Fold changes between the different conditions were calculated and visualized using a three-color scheme.

### EC adhesion and invasion assays

A total of 2.5 × 10^5^ Caco-2 or HEp-2 cells per well were seeded in a 24-well culture plate and cultivated for 48 h until infection. Cells were washed twice with PBS/Mg^2+^Ca^2+^ and covered for 35 min (adhesion assay) or 1 h (invasion assay) with 500 µl of DMEM containing approximately 2.5 × 10^6^ bacteria (multiplicity of infection, MOI, =10) from a glycerol stock washed with PBS. For glycerol stocks, strains were grown in 20 ml of BHI medium to mid-log phase (OD_600_ ~0.85–0.95) and supplemented with glycerol (15% final concentration). Aliquots of 1 ml were frozen at −80 °C. Prior to infection, samples were thawed, and the number of viable bacteria was determined as cfu per ml.

The average MOI was calculated immediately after infection and ranged from 8 to 11. For assessment of adhesion, the Caco-2 or HEp-2 cells were washed thrice with PBS/Mg^2+^Ca^2+^ after a 35-min incubation period. Cell layers were lysed in 1 ml of cold Triton X-100 (0.1%) and vortexed for 1 min to disrupt the cells. For invasion assays, Caco-2 or HEp-2 cells were washed twice with PBS/Mg^2+^Ca^2+^ after 1 h of incubation. Extracellular bacteria were removed by adding 0.5 ml of DMEM containing 100 μg/ml gentamycin for 1 h, and the medium was then replaced by DMEM with 10 µg/ml gentamycin. At appropriate time points of incubation in the presence of 10 μg/ml gentamycin, the infected eukaryotic cells were washed again with PBS/Mg^2+^Ca^2+^ and then lysed in 1 ml of cold Triton X-100 (0.1%) and vortexed. Adhesion and invasion characteristics as well as intracellular replication behavior of the mutants and the wild type were quantified by plating dilutions of the lysed cells on BHI agar plates that had ben incubated at 37 °C for one day. In all experiments, intact eukaryotic cell monolayers were observed prior to cell lysis.

### *In vitro* growth analyses

For growth analysis of *L. monocytogenes* EGDe and EGDe Δ*pduD*, we used the Bioscreen C Automated Microbiology Growth Curve Analysis System (Oy Growth Curves Ab Ltd., Helsinki, Finland), allowing automated OD measurement in a microvolume of 200 µl. Overnight cultures of *L. monocytogenes* grown in BHI at 37 °C were washed with PBS and then diluted in PBS to obtain an OD_600_ of 1. This cell suspension was further diluted 1:20 in MM^51^ containing 0.5% (w/v) yeast extract (Oxoid, Wesel Germany). MM was supplemented with 0 or 50 mM glucose (Fluka, Neu-Ulm, Germany). Cultures were incubated at 37 °C with continuous medium shaking (shaking steps: 60), and were overlaid with 200 µl of paraffin oil (Roth, Karlsruhe, Germany) to establish anaerobic conditions. The OD_600_ was automatically measured every 30 min over a period of 10 h.

### Mouse infections

Four-week-old female BALBc/By/J mice from The Jackson Laboratory (Bar Harbor, ME, USA) or BALB/cAnNCrl mice from Charles River Laboratories (Sulzfeld, Germany) were purchased and used for experiments at the age of 6-8 weeks. Mice were maintained in a specific-pathogen-free facility with a 14-h light and 10-h dark cycle. Mice were infected using a model for foodborne infection as described previously^46^. Mice were placed in a cage with raised wire flooring to prevent coprophagy and denied food for 18-22 h before the infection. Aliquots of frozen *L. monocytogenes* or other *Listeria* species were recovered in BHI medium for 1.5 h at 30 °C without shaking. The desired inoculum was resuspended in 2 µl of PBS mixed with 3 µl of salted butter (Kroger, Cincinatti, OH, USA or REWE, Cologne, Germany). Cell suspension was used to saturate a 2- to 3-mm piece of bread (Kroger or REWE). After the onset of the dark cycle, mice were transferred to an empty cage and fed the *Listeria*-contaminated pieces of bread with sterile forceps. Afterwards, the mice were returned to their raised wire flooring cages and mouse chow was replenished. Sample collection and handling was performed as described recently^46^. We confirm that all methods were carried out in accordance with relevant guidelines and regulations, and that all experimental protocols were approved by the Regierung von Oberbayern, München, Germany.

### Statistics

Statistical analyses for all experiments were performed using the Student’s *t*-Test with Welch’s correction in Prism6 (GraphPad, La Jolla, CA, USA). *P* values less than 0.05 were considered significant and are indicated as follows: *(P < 0.05); **(P < 0.01); ***(P < 0.001); NS (not significant, P ≥ 0.05).

## Electronic supplementary material

Supplementary material
