# Supplementary material for: Comparison between Listeria sensu stricto and Listeria sensu lato strains identifies novel determinants involved in infection
Source: Sci Rep. 2017 Dec 19;7:17821. doi: 10.1038/s41598-017-17570-0 (PMC5736727; doi:10.1038/s41598-017-17570-0)
Supplement: Supplementary file 1 — Supplementary material [file 41598_2017_17570_MOESM1_ESM.doc]

**Comparison between *Listeria sensu stricto* and *Listeria sensu lato* strains identifies novel determinants involved in infection**

Short title: *Listeria sensu stricto* specific genes

# Jakob Schardt^1^, Grant Jones^2^, Stefanie Müller-Herbst^1^, Kristina Schauer^3^, Sarah E. F. D’Orazio^2^ and Thilo M. Fuchs^1,4^*

*^1^ZIEL-Institute for Food & Health, and Lehrstuhl für Mikrobielle Ökologie, Wissenschaftszentrum Weihenstephan , Technische Universität München, Weihenstephaner Berg 3, 85354 Freising, Germany; ^2^Department of Microbiology, Immunology, & Molecular Genetics, University of Kentucky, Lexington, Kentucky, USA; ^3^Lehrstuhl für Hygiene und Technologie der Milch, Tiermedizinische Fakultät, Ludwig-Maximilians-Universität München, Schönleutner Str. 8, 85764 Oberschleißheim, Germany; ^4^Friedrich-Loeffler-Institut, Institut für Molekulare Pathogenese, Naumburger Str. 96a, 07743 Jena, Germany*

Keywords: *Listeria monocytogenes*, *Listeria* *sensu stricto*, oral infection, adhesion, 1,2-propanediol

*Corresponding author. Mailing address: Zentralinstitut für Ernährungs- und Lebensmittelforschung (ZIEL), Abteilung Mikrobiologie, Technische Universität München, Weihenstephaner Berg 3, D-85354 Freising, Germany. Phone: +49-8161-713859, Fax: +49- 8161-714492, E-mail: [thilom.fuchs@fli.de](mailto:thilom.fuchs@fli.de)

**Supplementary Material**

**Supplementary Fig. S1:** Female BALB/c mice were orally infected with 4-9 × 10^8^ cfu of the *Listeria sensu stricto* species *L. monocytogenes* (●) and *L. welshimeri* (■), as well as of the *Listeria sensu lato* species *L. aquatica* (▲) and *L. booriae* (▼). After 2 days, cfu numbers for each species were determined in the spleen, liver and MLN. Symbols represent values for individual mice, while horizontal lines indicate the mean value that was pooled from two separate experiments (n = 3 mice per group). Dashed lines represent the detection limit for each sample. Statistical significance was assessed using two-tailed student’s t-test with Welch’s correction.

**
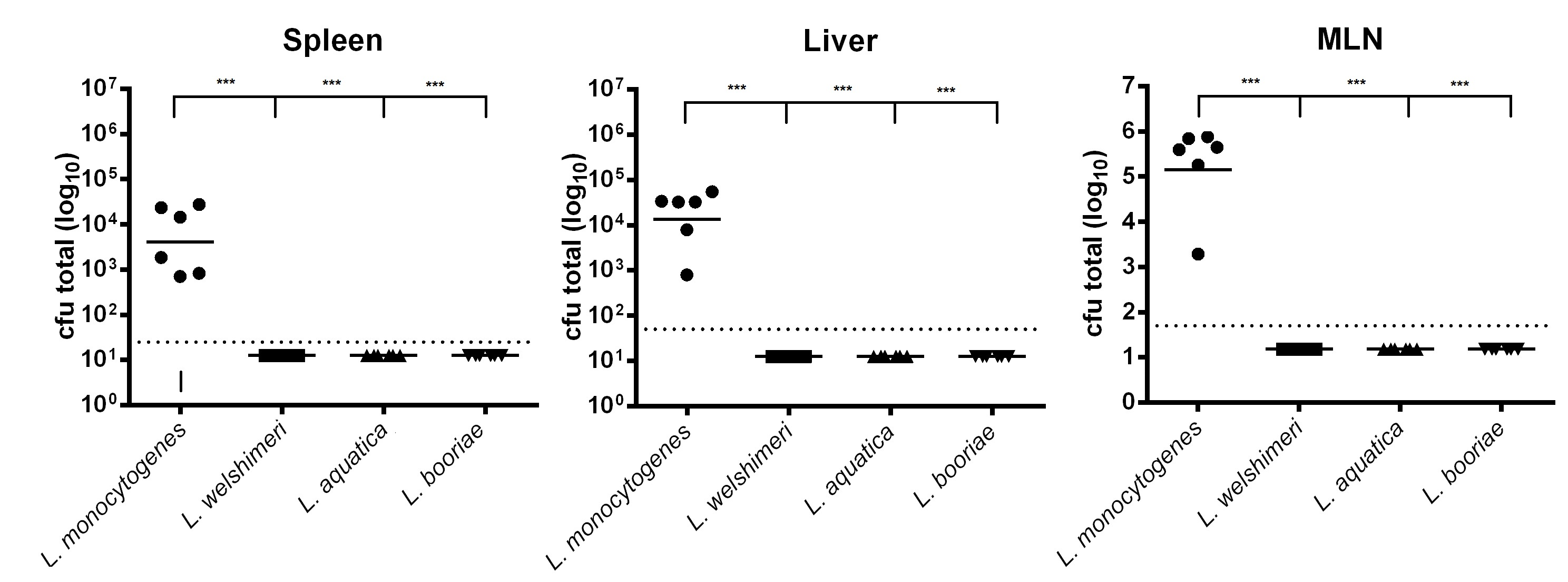
**

**Supplementary Fig. S2:** (a) Female BALB/c mice were orally infected with a 1:1 ratio of EGDe and EGDe Δlmo1131-1132 for a total inoculum of 1 × 10^9^ cfu. Two days p.i., the numbers of listeriae in the mucus, ECs and the LP of the ileum and the colon were determined. A pool of two separate experiments (n = 4 mice per group) is shown. (b) Female BALB/c mice were i.v.-infected with a 1:1 ratio of *L. monocytogenes* EGDe and EGDe Δlmo1131-1132 for a total inoculum of 1 × 10^4^ cfu. Liver and spleen were harvested 2 days p.i., and the cfu numbers of listeriae in these organs was analyzed. CIs depict the ratio of EGDe Δlmo1131-1132/EGDe. The geometric mean for each group was compared to the theoretical value of 1.0 and the fold change difference is indicated in parentheses.


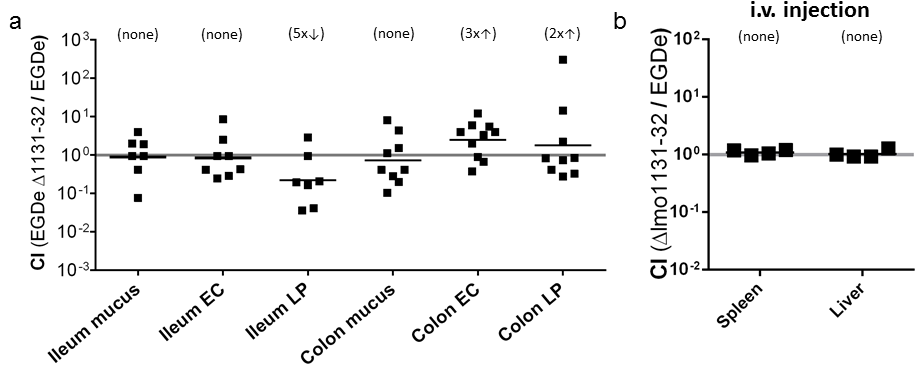


**Supplementary Table S1: Normalized RPKM values of *L. monocytogenes* EGDe for the conditions BHI, BHI with 10 mM 1,2-PD (BHI+1,2-PD) and BHI with 10 mM 1,2-PD and 25 nM cobalamin (BHI+1,2-PD+B_12_) during stationary and exponential phases.**

|  |  | **normalized RPKM values** | | | | | |  |  |  | **normalized RPKM values** | | | | | |
| --- | --- | --- | --- | --- | --- | --- | --- | --- | --- | --- | --- | --- | --- | --- | --- | --- |
|  |  | **Exponential Phase** | | | **Stationary Phase** | | |  |  |  | **Exponential Phase** | | | **Stationary Phase** | | |
|  | **Gene** | **BHI** | **BHI +1,2-PD** | **BHI + 1,2-PD + B_12_** | **BHI** | **BHI +1,2-PD** | **BHI + 1,2-PD + B_12_** |  |  | **Gene** | **BHI** | **BHI +1,2-PD** | **BHI + 1,2-PD + B_12_** | **BHI** | **BHI +1,2-PD** | **BHI + 1,2-PD + B_12_** |
| **Propanediol** | *pduS* (lmo1142) | 369 | 224 | 134 | 398 | 12,107 | 51,728 |  | **Cobalamine** | *cobU* (lmo1147) | 50 | 0 | 46 | 0 | 552 | 2,126 |
|  | *pduT* (lmo1143) | 173 | 176 | 79 | 322 | 5,638 | 24,056 |  |  | *cobS* (lmo1148) | 0 | 0 | 19 | 28 | 336 | 1,526 |
|  | *pduU* (lmo1144) | 37 | 262 | 117 | 99 | 1,463 | 5,883 |  |  | *cobC* (lmo1149) | 24 | 0 | 0 | 0 | 32 | 282 |
|  | *pduV* (lmo1145) | 0 | 39 | 24 | 52 | 798 | 2,916 |  |  | *cobD* (lmo1169) | 63 | 160 | 173 | 128 | 2,185 | 12,428 |
|  | *pduA* (lmo1151) | 0 | 0 | 22 | 0 | 1,070 | 7,810 |  |  | *cbiA* (lmo1191) | 12 | 0 | 0 | 58 | 1,984 | 17 |
|  | *pduB* (lmo1152) | 0 | 0 | 28 | 83 | 6,221 | 34,684 |  |  | *cbiB* (lmo1192) | 0 | 0 | 0 | 46 | 3,050 | 24 |
|  | *pduC* (lmo1153) | 20 | 41 | 36 | 81 | 10,014 | 62,732 |  |  | *cbiC* (lmo1193) | 0 | 0 | 0 | 24 | 2,984 | 0 |
|  | *pduD* (lmo1154) | 0 | 0 | 0 | 41 | 3,363 | 22,417 |  |  | *cbiD* (lmo1194) | 0 | 13 | 0 | 0 | 1,354 | 37 |
|  | *pduE* (lmo1155) | 0 | 26 | 11 | 0 | 2,414 | 15,558 |  |  | *cbiE* (lmo1195) | 0 | 0 | 0 | 13 | 1,277 | 13 |
|  | *pduG* (lmo1156) | 11 | 11 | 0 | 50 | 3,330 | 22,488 |  |  | *cbiT* (lmo1196) | 0 | 28 | 0 | 19 | 3,672 | 197 |
|  | *pduH* (lmo1157) | 0 | 0 | 0 | 0 | 587 | 3,713 |  |  | *cbiF* (lmo1197) | 0 | 0 | 0 | 21 | 1,554 | 78 |
|  | *pduK* (lmo1158) | 34 | 0 | 31 | 0 | 3,587 | 24,443 |  |  | *cbiG* (lmo1198) | 0 | 0 | 0 | 20 | 1,906 | 167 |
|  | *pduJ* (lmo1159) | 10 | 0 | 0 | 13 | 1,463 | 6,981 |  |  | *cbiH* (lmo1199) | 0 | 31 | 28 | 63 | 5,480 | 306 |
|  | *pduL* (lmo1160) | 0 | 0 | 0 | 137 | 6,123 | 28,847 |  |  | *cbiJ* (lmo1200) | 0 | 24 | 0 | 16 | 1,162 | 17 |
|  | *pduM* (lmo1162) | 64 | 130 | 29 | 130 | 5,527 | 32,136 |  |  | *cysG* (lmo1201) | 0 | 17 | 16 | 12 | 1,408 | 36 |
|  | *pduN* (lmo1163) | 0 | 0 | 0 | 75 | 1,256 | 8,371 |  |  | *cbiK* (lmo1202) | 0 | 25 | 11 | 0 | 600 | 17 |
|  | *pduO* (lmo1164) | 0 | 21 | 29 | 71 | 3,574 | 19,977 |  |  | *cbiL* (lmo1203) | 23 | 0 | 0 | 0 | 900 | 83 |
|  | *pduP* (lmo1165) | 0 | 37 | 50 | 50 | 5,801 | 28,766 |  |  | *cbiM* (lmo1204) | 0 | 0 | 0 | 8 | 359 | 17 |
|  | *pduQ* (lmo1166) | 0 | 0 | 31 | 136 | 10,731 | 58,994 |  |  | *cbiN* (lmo1205) | 0 | 0 | 0 | 15 | 955 | 79 |
|  | *pduF* (lmo1167) | 0 | 0 | 16 | 48 | 1,848 | 10,379 |  |  | *cbiQ* (lmo1206) | 0 | 0 | 0 | 17 | 511 | 0 |
|  | *pduW* (lmo1168) | 50 | 64 | 34 | 42 | 1,065 | 4,676 |  |  | *cbiO* (lmo1207) | 0 | 0 | 22 | 162 | 1,713 | 135 |
|  | *pduX* (lmo1170) | 125 | 356 | 160 | 102 | 378 | 3,741 |  |  | *cbiP* (lmo1208) | 417 | 604 | 515 | 1,929 | 7,500 | 2,178 |
|  | *pduL* (lmo1182) | 0 | 0 | 6 | 0 | 17 | 0 |  |  | *pocR* (lmo1150) | 32 | 0 | 72 | 150 | 391 | 847 |
|  | *pduT* (lmo1185) | 0 | 0 | 0 | 0 | 0 | 0 |  | **unknown** | lmo1146 | 32 | 32 | 58 | 65 | 1,047 | 3,250 |
|  |  |  |  |  |  |  |  |  |  | lmo1171 | 0 | 0 | 0 | 0 | 0 | 0 |
|  |  |  |  |  |  |  |  |  |  | lmo1172 | 65 | 0 | 30 | 66 | 45 | 92 |
|  |  |  |  |  |  |  |  |  |  | lmo1173 | 20 | 20 | 55 | 136 | 55 | 71 |
|  |  |  |  |  |  |  |  |  |  | lmo1183 | 0 | 126 | 28 | 0 | 0 | 175 |
|  |  |  |  |  |  |  |  |  |  | lmo1188 | 1,912 | 1,380 | 1,799 | 459 | 621 | 501 |
|  |  |  |  |  |  |  |  |  |  | lmo1189 | 205 | 80 | 158 | 32 | 108 | 166 |
|  |  |  |  |  |  |  |  |  |  | lmo1190 | 0 | 121 | 73 | 161 | 4,635 | 12,748 |

Supplementary Table S2. Strains and plasmids used in this study

| Bacterial strains | Description and relevant features | Source |
| --- | --- | --- |
| EGDe | *L. monocytogenes* Sv 1/2a, wild type (strain ATCC BAA-679) | G.B. Mackaness |
| DH5α | *E. coli: deoR* *endA1 gyrA96 hsdR17*(r_k_-m_k+_) *recA1 relA1* *supE44* *λthi-1* ∆(*lacZYA*-*argFV169*) | 1 |
| EGDe Δlmo1131-1132 | In-frame deletion of lmo1131-1132 in EGDe | This study |
| EGDe Δ*pduD* | In-frame deletion of *pduD* in EGDe | This study |
| EGDe::pIMC3kan | EGDe with pIMC3kan integrated at the tRNA^ARG^ | This study |
| EGDe Δlmo1131-1132::pIMC3ery | EGDe Δlmo1131-1132 with pIMC3ery integrated at the tRNA^ARG^ | This study |
| EGDe Δ*pduD*::pIMC3ery | EGDe Δ*pduD* with pIMC3ery integrated at the tRNA^ARG^ | This study |
| Plasmids |  |  |
| pLSV101 | Temperature-sensitive shuttle vector; erythromycin-resistant | 2 |
| pLSV101Δlmo1131-1132 | Deletion plasmid for lmo1131-1132 | This study |
| pIMC3kan | Site-specific integrative vector. IPTG controlled expression of *aphA*-III; chloramphenicol-resistant. | 3 |
| pIMC3ery | Site-specific integrative vector. IPTG controlled expression of *ermAM*; chloramphenicol-resistant. | 3 |

Supplementary Table S3. Oligonucleotides used in this study.

| Name of oligonucleotide (5´- 3´) | Sequence | Name of oligonucleotide (5´- 3´) | Sequence |
| --- | --- | --- | --- |
| construction of EGDe Δlmo1131-1132 | | qRT-PCR | |
| lmo1131-1132_A | TGCTTTTAATGGAAAGGGTCA | lmo1131_qRT_F | CACGATTAGTATGACTGGCA |
| lmo1131-1132_B | TTCAACGGTACCAAATAAACGCTTATCAATCAT | lmo1131-qRT_R | ACAATTTACTGCAAGCACTG |
| lmo1131-1132_C | GAAGAAGGTACCTTTATAGAAAATGGTGCATTG | lmo1132_qRT_F | TGAAAAGCTCAATAGGCAGT |
| lmo1131-1132_D | CGGCCAACTAATTCATTTGTA | lmo1132_qRT_R | GTTTCAAGTGGCAATTGTGA |
| lmo1131-1132_nestAB | GCGAATGTCGACTTTTTTGAATCGTTTTTTGAA | lmo1153_qRT_F | GGAGATGATACACCTTGGTC |
| lmo1131-1132_nestCD | GTAATGCCCGGGAATTTTTAATGTTTGTTATTG | lmo1153_qRT_R | TTTTCCTTCTGCATAACCCA |
| construction of EGDe Δ*pduD* | | lmo1199_qRT_F | AGCAACCCAGATTTAGAAGT |
| pduD_A | CGCACTCGGATATTCG | lmo1199_qRT_R | TTTCAATTACTTCCCACGGT |
| pduD_B | GAAGATCTT CTTTTTCGTTAATTTCAACC | lmo1190_qRT_F | GAATGATGGGCGTAACAATG |
| pduD_C | GAAGATCTTGTTCAAGGGAAAAACG | lmo1190_qRT_R | GTGCAAGTAATGATAATGGACA |
| pduD_D | GCCACTTCCGTTGTAG | lmo1150_qRT_F | TTTTCAGTCCCTATCGTTGT |
| pduD_nestedAB | CGGGATCCCGACGGTGGGTTAACAC | lmo1150_qRT_R | AAACGCGGAGATAAGATCC |
| pduD_nestedCD | CGGAATTCCGCTGTGCTGCTTCACG | lmo1146_qRT_F | ATGTTGTCGGAGGAGAAATG |
| pLSV101 specific primer | | lmo1146_qRT_R | CGCTGGTTTGAGTTACTTTC |
| LSV3 | AGTACCATTACTTATGAG | lmo1759_qRT_F | AAACGAACGGACAATTGAAG |
| LSV-4380rev | AGGGTTTTCCCAGTCACG | lmo1759_qRT_R | CTAACTTATCGACGTCAGCA |
|  |  | 16S_R | AGACACGGCCCAGACTCCT |
|  |  | 16S_F | GATCCGAAAACCTTCTTCATACA |

References

1 Hanahan, D. Studies on transformation of *Escherichia coli* with plasmids. *J Mol Biol* **166**, 557-580 (1983).

2 Joseph, B. *et al.* Identification of *Listeria monocytogenes* genes contributing to intracellular replication by expression profiling and mutant screening. *J Bacteriol* **188**, 556-568 (2006).

3 Monk, I. R., Casey, P. G., Cronin, M., Gahan, C. G. & Hill, C. Development of multiple strain competitive index assays for *Listeria monocytogenes* using pIMC; a new site-specific integrative vector. *BMC Microbiol* **8**, 96 (2008).
